# Behavioural responses of white sharks to specific baits during cage diving ecotourism

**DOI:** 10.1038/s41598-020-67947-x

**Published:** 2020-07-07

**Authors:** Edgar E. Becerril-García, Edgar M. Hoyos-Padilla, Primo Micarelli, Felipe Galván-Magaña, Emilio Sperone

**Affiliations:** 1 Instituto Politécnico Nacional, Centro Interdisciplinario de Ciencias Marinas, 23096 La Paz, Mexico; 2 Pelagios Kakunjá A.C., 23060 La Paz, Mexico; 3 Fins Attached Marine Research and Conservation, Colorado Springs, 80908 USA; 4 Sharks Studies Center, 58024 Massa Marittima, Italy; 5 Department of Biology, Ecology and Earth Sciences, University of Calabria, 87036 Rende, Italy

**Keywords:** Marine biology, Animal behaviour, Ichthyology, Behavioural ecology, Conservation biology

## Abstract

This study describes the effect of different baits on the attraction, surface behaviour and conditioning of white sharks *Carcharodon carcharias* during local ecotourism activities. The sightings, behaviours, and pictures used for photographic identification were obtained during August to November 2012–2014 onboard tourist boats in Guadalupe Island, Mexico. Four types of baits were used: (1) frozen bait; (2) frozen bait and natural chum; (3) fresh fish bait; and (4) mackerel bags. Data were analysed according to sex, maturity and the total of sharks using 6,145 sightings of 121 white sharks. The type of bait showed no significant difference on the effectiveness to attracting sharks. Ethological analysis showed that the type of bait had a significant effect on the shark’s surface behaviour during its interactions with boats. Natural chum and fresh baits showed short term behavioural patterns constituted by increased number of violent interactions with the bait, while the frozen bait did not generate a defined behavioural pattern. Conditioning of white sharks was determined by the number of interactions and the consumption frequency of the bait. Fifty nine percent of sharks (n = 41) showed no conditioning, 36% (n = 25) showed a low risk and only 5% (n = 3) were found to have a high risk of conditioning. The results suggest that current ecotourism has no effect on the conditioning of the white sharks, and that all baits have a similar effectiveness for attracting the sharks. However, a different behavioural pattern was observed when fresh bait and chum were used, which could increase the potential of accidents during ecotourism.

## Introduction

Ecotourism with white sharks *Carcharodon carcharias* (Linnaeus, 1758) occurs in countries where seasonal aggregations of this species have been observed, including South Africa^1^, Australia^2^, New Zealand^3^, the United States of America^4^ and Mexico^5^. Due to national and international wildlife regulations, shark cage diving is currently the main legal activity for the economical use of this vulnerable species^1,6^. In Mexico, cage diving began in 2001 with the identification of Guadalupe Island as one of the main aggregation sites for *C. carcharias* in the Eastern Pacific Ocean^5,7^. Since then, numerous ecotourism operators work between August and November of each year, also providing on going surveillance against illegal fisheries, and a platform for both public education and scientific research. The local revenue of cage diving in Guadalupe has been estimated at more than 4.5 US million dollars per season^8,9^.

Regardless of the benefits, white shark ecotourism is often seen as a controversial subject. In the case of cage diving, provisioning of food has been linked with potential negative effects on habitat use, surface behaviour, bioenergetics, conditioning and a probable increase in the frequency of interactions with humans^1,2,10,11^. In Guadalupe Island, the study of the effects from ecotourism is a priority for local authorities and for the conservation of this threatened species^11^. Since 2016, there have been at least six accidents related to cage diving in this oceanic island, as some white sharks and divers have been injured during these events^8,9^. However, the most serious accident occurred in October 2019, when a white shark died after being stuck in a cage for more than 25 min^12^. In this regard, specific violent behaviours related to the capture of bait increase the risk of such accidents, in which these behaviours have been described until now as “attacks”^8,12,13^.

In terms of management, the evaluation of the attraction, surface behaviour and potential conditioning of the sharks is needed for regulations related to the use of bait and for the prevention of accidents during cage diving^8^. For the activities conducted in this marine protected area, only frozen fish (*Thunnus albacares*) bought in the departure port is allowed as bait during ecotourism. Nevertheless, some operators often use fresh bait for cage diving activities, in the belief that the olfactory cue from a fresh fish could attract sharks more efficiently.

The aim of the present study is to describe the effect of different baits on the attraction, surface behaviour and conditioning of white sharks during ecotourism in order to provide information for the sustainable use, economical exploitation and the prevention of accidents between white sharks, cages and divers. Beyond the concept of conditioning that could negatively affect the ecology of sharks involved in ecotourism, our study aims to test the hypothesis that the type of bait used to approach sharks has an influence on their surface behaviour. The implications for conservation include the concerns of authorities, cage diving operators, local fishermen, tourists, and scientists for improving the management of this vulnerable and iconic species.

## Results

### Attraction to bait

**Figure 1:**
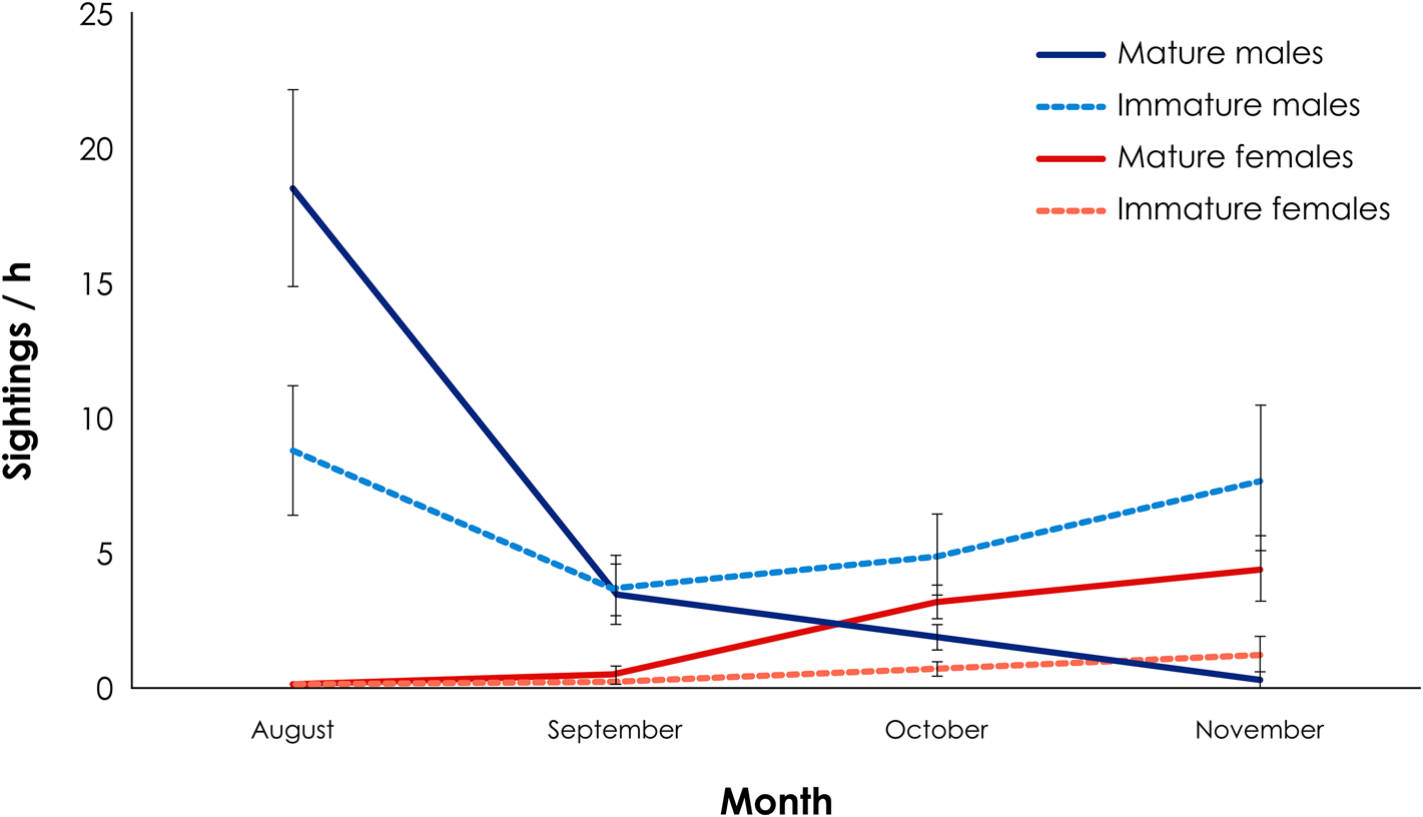
Average of sightings per hour and standard error according to sexual maturity and month during the seasons 2012–2014 in Guadalupe Island, Mexico.

A total of 6,145 sightings from 121 identified white sharks were registered during 87 days at sea. Seventy four percent of these sightings were males and 26% were females, which showed a significant sexual proportion of 3:1 (M:F; P < 0.01). In general, there were no statistical differences between maturity stages along the studied months, as 51% were mature and 49% were immature individuals (P > 0.05). However, the presence of white sharks changed during the season. A maximum average of sightings per hour of mature males was observed during August, with a decrease of this number further in the season and an increase of mature females since late September (Fig. 1).

The general presence of white sharks was similar regardless of the type of bait. A maximum of 70.5 sightings per hour was registered during the use of frozen bait and chum, while the minimum 0.17 sightings per hour was observed using mackerel bait bag. Nonetheless, this minimum and maximum data were outliers and did not affect the lack of significant differences of sightings between the types of baits (H_3,87_ = 8.29, P > 0.05). A similar effect was observed when sightings were analysed by sexual maturity stage and the type of bait. There were no significant differences in the sightings per hour of mature males (H_3,87_ = 5.91, P > 0.05), mature females (H_3,87_ = 1.88, P > 0.05), immature males (H_3,87_ = 17.05; P > 0.05) or immature females (H_3,87_ = 7.40, P > 0.05) between the four baits that were tested.

### Surface behaviour

Ethological analyses were carried out using data from 534 h of direct observation of white sharks that interacted with the tourist boats. This monitoring allowed to obtain 17,360 files of audio-visual material, which was filmed by the observer or provided by the tourists who visited Guadalupe Island during the study period. For these analyses, only the data from 106 different sharks were used (n = 4,823 behaviours), as the ethograms from 15 sharks were influenced by the presence of other individuals.

According to the category, a total of 21 mature males, 18 mature females, 54 immature males and 13 immature females were photo identified during the study period. The size range for both sexes was 1.8–5.8 m TL with a mean of 3.6 m ± 0.9 m. The mean size of males was 3.3 m ± 0.7 cm TL, and 4.2 ± 1.1 m TL for females. Regarding individuals, a significant sex ratio of 1:2.4 was observed (H: M, p = 5.542e−5); with 63% of immature individuals and 37% of mature sharks. A total of 1,542 ethograms of the surface behaviour of white sharks were generated (Table 1).

**Table 1:** Number of ethograms according to white shark group and type of bait in Guadalupe Island during 2012–2014 seasons.

Group	Frozen bait	Frozen bait and chum	Fresh bait	Mackerel bag	Total
Male	67	218	247	6	538
Females	37	92	104	0	233
Adults	48	209	178	0	435
Juveniles	56	101	173	6	336
Total	208	620	702	12	1,542

Success rate for the capture and consumption of bait was similar in all categories, although a higher rate for male sharks and mature individuals was observed. Proportionally, a mature shark would get the bait 22 times for every 100 foraging attempts. Consumption rate was lower than the catch rate, as sharks did not always consume the captured bait. Similarly, mature sharks presented the highest amount of consumption in relation to the number of foraging attempts made (Table 2).

**Table 2:** Effectiveness of white shark attacks by group during ecotourism in Guadalupe Island, Mexico.

Group	Bait capture rate (%)	Consumption rate (%)
Male	20	12
Females	17	10
Mature	22	14
Immature	17	10

**Figure 2:**
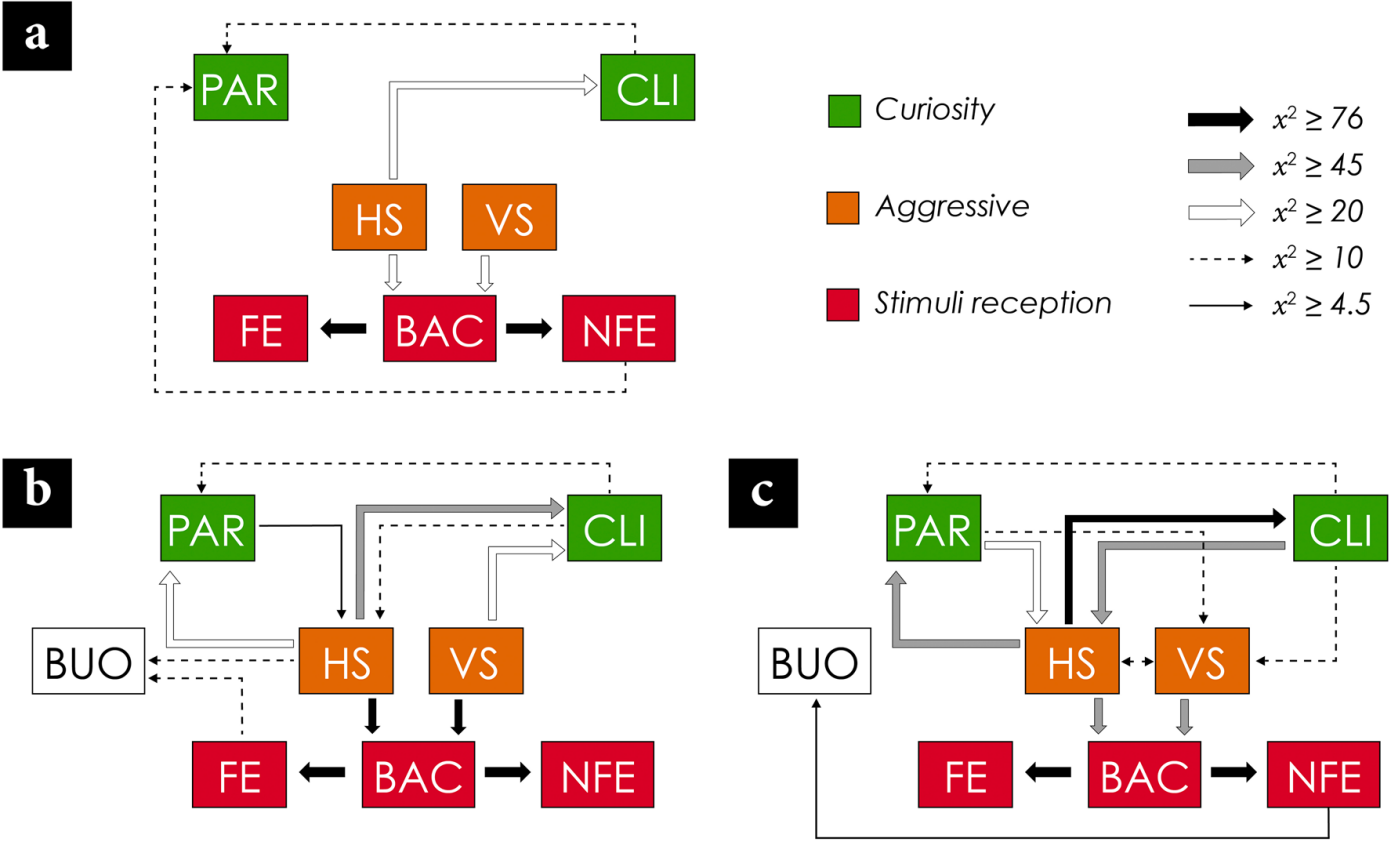
Ethological diagrams with the significant transitions (black arrows) between the observed behaviours according to the type of bait, including (**a**) Frozen bait (n = 208 ethograms); (**b**) Frozen bait and chum (n = 620 ethograms); and (**c**) Fresh bait (n = 702 ethograms). Parading (PAR); Close Inspection (CLI); Horizontal Attack (HA); Vertical Attack (VA); BAC (Bait caught); FE (Feeding); NFE (No feeding).

The behaviour of white sharks was significantly different depending on the type of bait. Nevertheless, the bait known as mackerel bag was not considered for the ethological analysis, given that the boat that used it changed the bait early in the study period and there were not enough observations for the analysis. During the provisioning of frozen bait, the behavioural transitions from horizontal strikes (HS) to close inspections (CLI; χ^2^_49_ = 21.2, P > 0.05), vertical strikes (VS) to bait capture (BAC; χ^2^_49_ = 35.4, P > 0.05) and HA to BAC (χ^2^_49_ = 22, P > 0.05) were frequently observed, but with no significant contributions. The lack of statistically significant behaviour transitions showed that there was no specific behavioural pattern for the consumption of this type of bait (Fig. 2a). However, this pattern differed when chum was provided.

The interaction with frozen bait and chum showed a statistically significant transition from vertical (χ^2^_49_ = 105.1, P < 0.05) and horizontal strikes (χ^2^_49_ = 112.9, P < 0.05) towards BAC and feeding (FE; χ^2^_49_ = 679.8, P < 0.05). Although there were interactions from HA to CLI (χ^2^_49_ = 46.65, P > 0.05), and from HA to parading (PAR; χ^2^_49_ = 33.68, P > 0.05), there was no statistical significance on such transitions. During these situations, the behavioural pattern consisted in agonistic behaviours for the bait capture with constant repetitions of the same displays (Fig. 2b).

Regarding the fresh bait, only one significant transition from HA to CLI was observed (χ^2^_49_ = 95.19, P < 0.05). The transitions from CLI to HA (χ^2^_49_ = 49.19, P > 0.05), HA to PAR (χ^2^_49_ = 53.29, P > 0.05) and from horizontal (χ^2^_49_ = 45.89, P > 0.05) or vertical strikes to BAC (χ^2^_49_ = 48.53, P > 0.05) were frequently observed; however, there was no statistical significance in such transitions (Fig. 2c). This pattern was identified for the agonistic behaviours and curiosity with close inspections to the bait, but with no significant transitions for feeding. Additionally, some displays were repeated constantly before they could perform any transition to another behaviour.

### Conditioning

The conditioning was determined on 69 photo identified white sharks, in which its identity was confirmed using high-quality images over the study period. These individuals correspond to 57% of the total recorded sharks (n = 121), as the remaining sharks had a deficient photographic record that did not allow the confirmation of the identity in the recaptures. The decision to use only high-quality photographs was made in order to avoid overestimation of the number of white sharks for the conditioning analysis.

**Figure 3:**
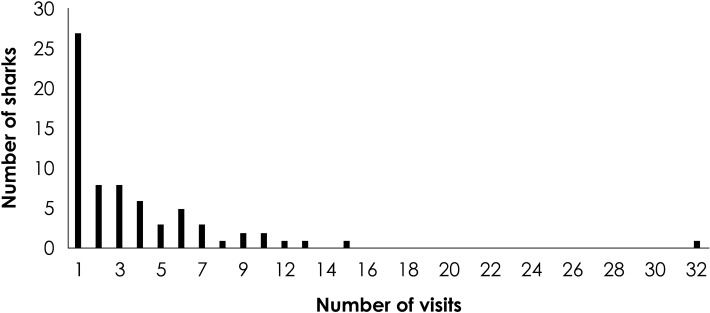
Frequency of visits recorded from the monitoring of 69 photo identified white sharks that interacted with the tourist boats during 2012–2014 in Guadalupe Island, Mexico.

An average of 2 ± 5 daily visits per white shark was recorded during the study period. A total of 35 sharks (50.7%) visited the boats less than two times, while only one shark visited the tourist boats in 37 occasions (Fig. 3). According to the Spearman correlation test result, the number of sharks decrease with the increment in the number of visits (R = 0.82; P < 0.01).

**Figure 4:**
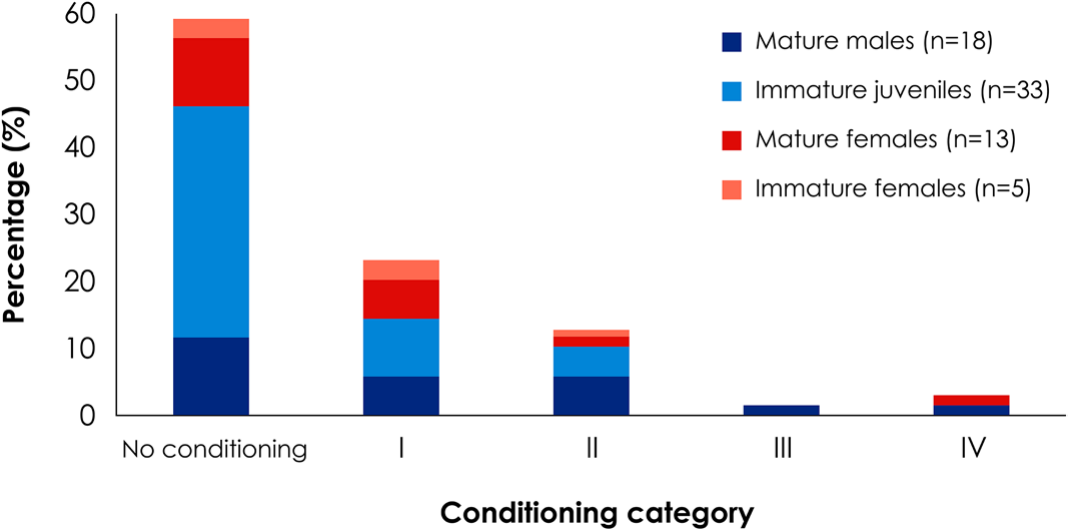
Percentage of identified white sharks according to their conditioning degree (n = 69) during the 2012–2014 seasons in Guadalupe Island, Mexico.

Most of the white sharks showed no signs of conditioning to the bait, as 59% of the sharks (n = 41) were registered in less than three random days in the season (Fig. 4). Twenty two percent were included in category I, since they corresponded to sharks that visited the vessels more than three random days per season (n = 16). Thirteen percent were classified in category II, which included sharks that visited the vessels for 3–5 days per month (n = 9). A single individual (1.44%) was catalogued in category III by being sighted more than 5 days per month and consuming bait during such periods. Finally, two of the sharks (2.88%) were included in category IV, as they occurred frequently, interacted with the boats more than 20 min per day and fed from the bait. Regarding sexual maturity, only mature individuals had a high grade of conditioning (categories III and IV), while the lower conditioning included both mature and immature sharks. The immature males were the most numerous sharks without conditioning (43.78%) in category I (8.69%) and category II (4.39%). Immature females were the least numerous among the conditioning categories analysed, while mature individuals, both males and females, were classified with a similar proportion in all cases (Fig. 4).

## Discussion and conclusions

The surface presence of white sharks was similar regardless maturity, sex, or type of bait, suggesting a similar effectiveness in the attractant of sharks. In other ethological studies, the white shark has been suggested as a predator that evaluates the consumption of food through previous inspection and monitoring^14–16^. In this instance, the use of frozen or fresh bait had a similar effectiveness when attracting sharks, mainly due to the visual and olfactory stimuli inherent to the bait^16–18^. It is possible that the scent of the frozen bait was not significantly different from a fresh bait, since there is no nutritional differences between both treatments^19^. This has been observed in other pelagic species such as the yellow fin tuna *T. albacares*, where the catches are similar regardless of the freshness of the bait^20^. However, the smell would be the main sense involved in the attraction at distance, while the sight would be fundamental in the decision of the capturing of prey^20,21^.

In other aggregation sites, the use of bait has shown a minimum effect on the white sharks behaviour^2,3^. In South Africa, Laroche et al*.*^1^ suggest that the population of 64,000 Cape fur seals, *Arctocephalus pusillus pusillus*, generates a constant olfactory stimulus for the white sharks, so the use of bait is negligible for the attraction and conditioning of *C. carcharias*^1,22^. A similar effect occurs in Farallon Islands where cage diving is often difficult, probably explained by the presence of a high density of northern elephant seals *Mirounga angustirostris*^4^. The extension of both sites is 0.02 km^2^ for the South African site, and 0.42 km^2^ for the American one. If it is compared with the 241 km^2^ of Guadalupe Island, the density of pinnipeds could be much lower in this oceanic island. In this regard, the absence of a natural, intense, and permanent stimulus in Guadalupe Island could explain both the success to attract sharks to the boats and the inherent risk of conditioning.

The idea that a fresh stimulus could generate a greater number of sightings cannot be accepted according to the results of the present study, since all the baits showed a similar effect on the attraction of the sharks. This lack of preference for a particular attractant may be related to the opportunistic predation on dead animals such as whale carcasses that have been observed in mature and immature white sharks^23–26^. Future analysis involving the hour of arrival of each shark according to the type of bait could be useful to determine the effectiveness in terms of time, which could complement the information for boat owners during ecotourism.

There were significant differences in the observed behavioural patterns according to the type of bait. The behavioural patterns with the frozen bait with chum and fresh bait were related to the reception of stimuli and feeding, while the frozen bait did not generate a defined pattern. It is possible that the smell and appearance of a frozen bait generate an olfactory and visual attraction in the white sharks without aggressions^16,21^. In contrast, the use of fresh bait along with the stimuli of a recently captured animal could be part of the explanation for the violent behaviours observed with this type of bait^14,17,18^.

Contrary to the pattern observed with the use of fresh bait, the behavioural transitions towards aggressive displays were not detected during the use of a frozen bait. This lower number of agonistic displays could decrease the risk of accidents of white sharks that enter or hit the cages, as it is frequently observed during each cage diving season^22^. However, vigilance and specifications for the handling of the bait, as well as for the redesign of the cages are the first steps to reduce the incidence of accidents^11,12,22^.

The horizontal strike (HS) was the most frequent behaviour during the study period, as it has been similarly observed in Australia by Tricas^27^ and Strong^14^, and in South Africa by Sperone et al*.*^15^. The large number of foraging attempts with respect to the number of captures and consumptions was the cause of the low effectiveness of this behaviour. In comparison with the immature individuals, the mature sharks managed to capture and consume the bait more efficiently. This may be attributable to the experience and size of white sharks according to their age, since mature sharks perform a better evaluation of the conditions before ambushing their prey to efficiently manage their energy when compared to immature individuals^22,28^.

In previous research, a low effectiveness of the foraging attempts has been related to energy loss and a physiological imbalance that could affect the health of the sharks^10,11,22^. An evaluation of the effects on the metabolic rate of the white sharks could provide insights in terms of energy loss caused by ecotourism, as it has been observed in other species of sharks^29,30^. However, an energetic affectation is unlikely if there is a low conditioning in the sharks that interacted with the boats, as it was observed under the current ecotourism conditions of Guadalupe Island.

The development of a conditioning to the boats requires defined patterns for feeding, since the behaviours and stimuli must be constant for this learning^1,31–33^. The sharks that interacted with the frozen bait presented a diverse number of behaviours for the acquisition of food, so this high variability of displays would prevent the development of conditioning in the absence of a defined behavioural pattern. In contrast, chum and fresh baits did demonstrate a pattern directed toward aggressions and reception of stimuli. Unlike those observed by Laroche et al*.*^1^, it is possible that the baits such as chum or fresh tuna could generate a future change in the behaviour of *C. carcharias* in Guadalupe Island^22^. In this regard, trophic interactions of each white shark population should be considered for future comparative studies, as differences in diet could be involved in the response and conditioning to the used baits^6,9^.

It is improbable that the ecotourism generates a conditioning on the white sharks of Guadalupe Island. Although the used criteria underestimate the cognitive capacity of the white shark, there are five arguments that may explain the lack of conditioning in this aggregation site.

1. Few visits of sharks. In other studies, conditioning was observed after 6 months of interaction with the constant provision of substantial food in training sessions that lasted more than 20 min^32,34^. In this study, most of the sharks were recorded in less than 5 days, so their low permanence would not allow a conditioning^32,35,36^.
2. Duration of the season. Studies in captivity have shown that conditioning can persist after 10 weeks in the memory of sharks^31^. This conditioning arises after receiving a constant stimulus under controlled situations^31,32,36^. In Guadalupe Island, the stimuli granted by their natural prey availability^5,37^, the white shark migration habits and its local movements^5,38^, as well as the 8 months between seasons^11^ could affect the duration of conditioning in terms of the memory of the sharks^31,32^. Under this assumption, the shark’s attraction to the boats would be the result of a natural curiosity and not of acquired learning^16,17,39^.
3. Low consumption of bait. In previous studies, the reception of "substantial" stimuli was one of the criteria necessary to determine a high risk of conditioning^31,32,36^. In the present research, the capture of at least one bait was considered as substantial and significant for the detection of conditioning in a precautionary measure to detect any indication of possible conditioning. Despite this, it was not possible to detect more than three sharks that fulfilled the bait intake, the number of visits and necessary interactions to determine a high risk of conditioning.
4. Effectiveness. The effectiveness of the strikes or foraging attempts was low for the capture and consumption of bait, so this lack of stimuli could lead to the disappearance of the potential acquired response in conditioning. White sharks from Guadalupe did not have many reinforcements, and the amount of strikes in means of energy used to capture them may not be profitable for their energetics^22^. A conditioning is unlikely when not enough stimuli is received during the development or maintenance of learning^31,33^.
5. Competition. The highest number of sightings was registered during the month of August and corresponded mostly to mature and immature males. It is possible that the presence of sharks of similar size favours the intraspecific interactions with respect to the bait, due to a greater tolerance on the part of the males of similar sizes^22^. However, the decrease in sightings during the following months could be related to the presence of larger sharks, which, given a hierarchy by size, would displace small sharks that could be more vulnerable to conditioning. In this manner, the segregated migration of mature sharks, the size hierarchy and competition would prevent immature individuals from developing a conditioning^11,22,38^.

In any case, the learning capacity of sharks could be higher during their juvenile stages^32,34^, so the monitoring of these individuals is essential to understand the possible changes in their presence or behaviour. A high number of immature males were observed during all season, so the vigilance in order to avoid the consumption of bait would represent a measure of precautionary management to prevent the conditioning of the white sharks^11,22,32^.

The study of the behaviour of sharks is necessary in the localities were ecotourism occurs. Although some activities like snorkelling, scuba diving or cage diving are considered as sustainable, there are few studies related to the negative effects of ecotourism in most shark populations^2,29,30,40–42^. The acquisition of data through science-based monitoring is a practical strategy that could improve the knowledge about the biology of the species, its relations with ecotourism and the correct decisions for good practices^22^. Some of the proper management regulations could include tourism seasonality, quantity of boats and the responsible use of organic attractants if these were needed^9,22^.

As a cosmopolitan species that is economically exploited in several countries, the presented results of the behaviour of white sharks could be useful in future comparisons with other populations^6,22^. This observational method is applicable without the need of invasive techniques or high economical resources, which could facilitate the obtention of data through the participation of trained observers and the tourism inclusion in scientific activities. This could lead to a significant contribution to local marine policies for efficient protection and sustainable use of the sharks, as it has been observed in other marine protected areas^43,44^.

Wildlife regulations during ecotourism are essential for the prevention of accidents between humans and sharks. The limited vigilance in Guadalupe Island, along with a lack of scientific evidence supporting some of the unfollowed regulations, were part of the cause for several accidents that occurred in 2016 and the years before, where several sharks and divers were harmed during ecotourism^44^, including the recent death of a white shark in 2019^12^. Although these situations are scarce and could not be avoided in some cases, the information regarding the behaviour to specific type of baits is needed for the prevention of accidents through science-based decisions. In the present paper, the different short-term surface behaviours according to the type of bait are provided in order to prevent more accidents, as well as to improve the monitoring of this threatened species.

The creation of a standard governmental monitoring using the presented methods will be useful for the constant evaluation of this and other shark populations. Future studies should consider the effect of the environment, social behaviour, personalities of sharks, times of response to the bait and movements of the sharks by the use of other techniques such as drone video and acoustic tagging in relation to the boats, which could provide more insights about the effects of cage diving ecotourism^2,42^. Additionally, the use of genetics and other photo identification methods, such as the analysis of dorsal fins and mark-recapture studies, would be beneficial for the knowledge of the status of this white shark population^45,46^.

The improvement of monitoring through the participation of trained observers would allow the generation of scientific knowledge in terms of ecology, demography and ethology if the proper data are frequently obtained^9,22,27^. Research involving minimum invasive techniques as the one presented in this paper could be useful for the generation of such information, which in turn can be used for the improvement of the activity in terms of sustainability and the conservation of this threatened species. Specific recommendations for cage diving ecotourism could be the creation of emergency response protocols for stuck sharks and injured divers, as well as the implementation of a nautical video vigilance cameras onboard all boats. The latter could be useful for the governmental inspection of good practices and the obtention of data in one of the most profitable and sustainable activities with sharks worldwide^6,9^.

## Materials and methods

### Study area

**Figure 5:**
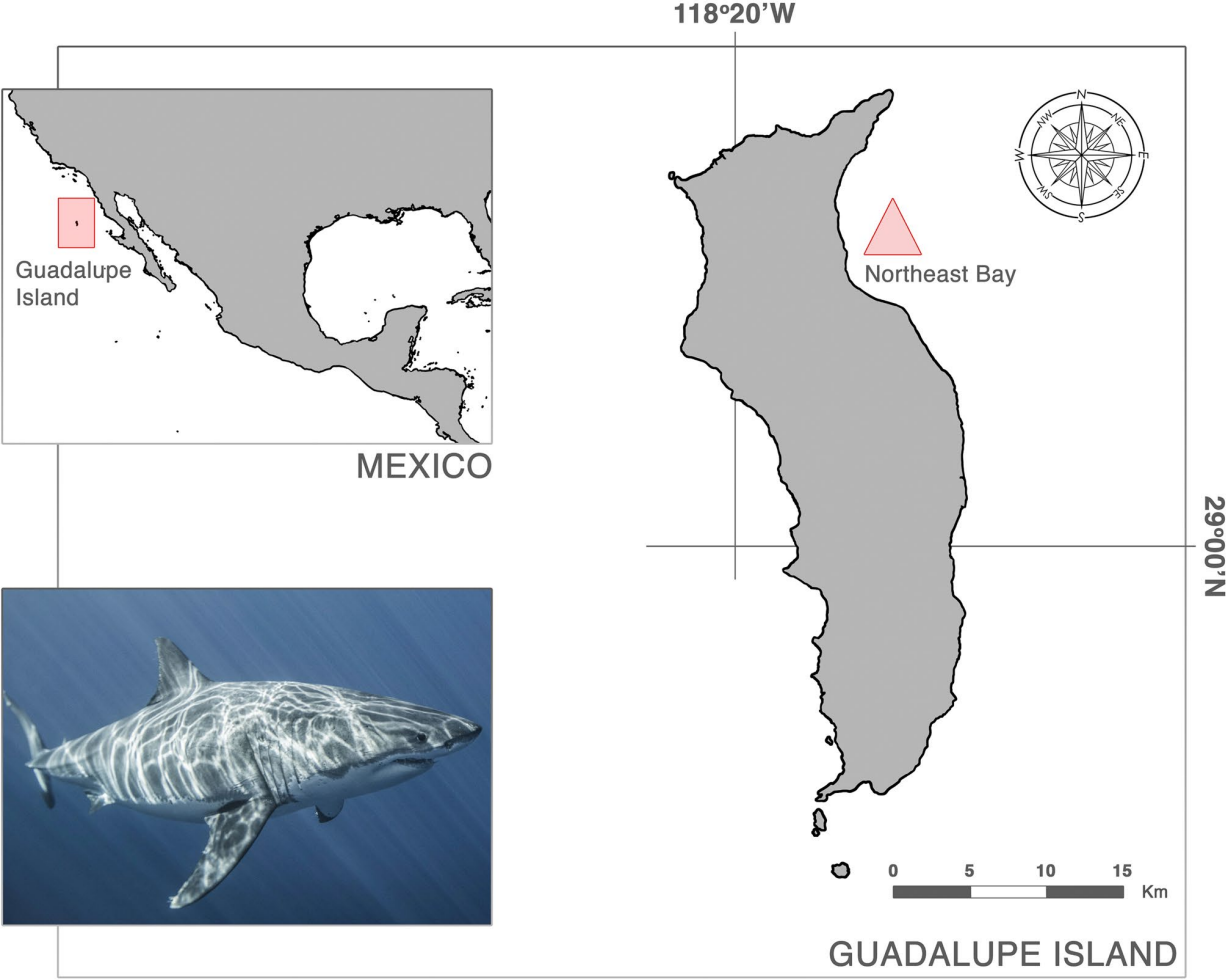
Location of Rada Norte Bay (filled triangle) where cage diving with white sharks occurs in Guadalupe Island, Mexico.

The Guadalupe Island Biosphere Reserve is a marine protected area located in the Pacific Ocean, 241 km offshore Baja California, Mexico (Fig. 5). It measures 32 km long and 6.5–9.5 km wide, with a total area of 244 km^2^ and a maximum elevation of 1,295 m^47^. These dimensions make Guadalupe Island the largest aggregation site for white sharks compared with other islands where this species occurs. However, the designated area for cage diving in Guadalupe Island is limited to a bay known as Rada Norte, which measures 7 km long and 2 km wide^11^.

In this volcanic island, the dominant ecosystems are sandy bottoms and rocky reefs with macroalgae forests, characterized by a high abundance of invertebrates, bony fishes, elasmobranchs, cetaceans and pinnipeds^37,47^. This biodiversity has been related to the seasonal presence of white sharks in terms of prey availability, as confirmed by observed predation events and stable isotopes analyses^5,47,48^.

The ecotourism boats surveyed during the study period were seven long-range vessels that measure 27–41 m in length. Boats were anchored at 200–250 m off the coast at a depth of 70–80 m, with two surface cages and a maximum capacity of four people each. Cage diving operations were supervised by the captain, a dive master and two crew members who were responsible of handling the bait and not feeding the sharks intentionally, as indicated by local regulations^11^.

## Data collection

In this study, all observations were carried out by one of the authors (EEBG) in order to maintain the bias of estimations in the same observer. The methods were approved under the following permits: Secretaría del Medio Ambiente y Recursos Naturales (SGPA/DGVS/06302/12; SGPA/DGVS/05847/13; SGPA/DGVS/03077/14), Comisión Nacional de Áreas Naturales Protegidas (F00.DRPBC.RBIG.‐210/12; F00.DRPBCPN.‐000889; F00.DRPBC.RBIG. 163/14) and Secretaría de Gobernación (SATI/PC/039/12; SATI/PC/038/13; SRPAP/PC/022/14).

Data were recorded on board the tourist boats that visited Guadalupe Island during August to November of 2012–2014, with a daily monitoring of white sharks between 07:00 and 18:00 h. Date, sex, total length (TL), type of behaviour, type of bait and the time of each shark sighting were recorded. The sex of each shark was determined by the presence or absence of claspers, with posterior confirmation through underwater photography. The TL was estimated using the length of the cages as a comparison with the length of the sharks when each shark approached horizontally, parallel, and close to the cages.

Identification of shark individuals was carried out by analysing underwater pictures based on several characteristics such as skin pigmentation, patterns of the gills slits, pelvic fins and caudal fin of both sides of the body, scars, mutilations, dorsal fins and anatomical deformities observed on the sharks using a Nikon D7100 camera inside an Ikelite Housing; additional photographs were voluntary provided from tourists. All pictures were analysed the same day that they were obtained to determine the identity of the white sharks that visited the boats. Sexual maturity was determined according to the sex and length, where males with a TL > 3.5 m and females with a TL > 4.5 m were considered mature individuals^24^. Shark sightings were posteriorly classified according to their sex and maturity stage. In this study, the analysed groups and statistical units for each analysis were specified for the objectives of attraction (average of sightings per hour), surface behaviour (ethograms from different individuals), or conditioning (photo identified white sharks), with respect to one of the following types of bait:

1. Frozen bait. Head, tail or any other segment cut from yellow fin tunas (*T. albacares*) acquired in the departure port. This piece of fish was tied to a rope and a buoy for flotation.
2. Frozen bait and chum. Frozen tuna accompanied by organic shedding known as chum, which is composed of a mixture of seawater with fish tissues such as blood, muscle, skin, fat, bone, etc.
3. Fresh bait. Pieces of fresh yellowfin tuna that were tied to a rope and a buoy for flotation; obtained in the area during tourist activities.
4. Mackerel bag. Pieces of mackerel (*Scomber japonicus*) placed in an organic fibre bag and tied to a line with a buoy.

### Attraction to bait

The average of sightings per hour was the statistical unit for analysis of the attraction of white sharks related to the type of bait in order to describe the effectiveness of each stimuli during ecotourism. A sighting was defined as any behaviour that an observed shark performed on the surface at a radial distance of 10 m with respect to the position of the bait. Since the data set did not meet the normality assumptions according to the Lilliefors test (n = 87; d = 0.14358, P < 0.01), a nonparametric Kruskal–Wallis test and a Bonferroni test were carried out for the analysis of such sightings per hour and bait according to the following categories: immature males, mature males, immature females and mature females.

### Surface behaviour

White shark behaviour was recorded in ethograms based on sighting frequencies and behaviour sequences that were further included in ethological flow charts for the representation of behavioural patterns in relation to the type of bait. Sightings were classified using the behaviours observed in the ethological analysis of baited attracted white sharks by Becerril-García et al*.*^22^, and the methods published by Klimley et al*.*^4^, Martin^15^ and Sperone et al*.*^16,39^. In an effort to use a less connoted terminology, the present study will use the neutral term of “feeding strike” or “feeding attempts”^13^. In this regard, a biological interpretation was used in order to classify each behaviour and to provide useful management information (Table 3). During the analysis, only the ethograms of white sharks in the absence of other sharks were considered, due to the possibility of intraspecific interactions affecting their behaviours with the bait. For the construction of the behavioural diagrams, the frequencies of each observed behaviour and transition were analysed using the software EthoLog v. 2.25^49^. The statistical significance of such transitions was determined through a Chi‐square test under the null hypothesis that there was no relationship between independent behaviours^16,39^. In this regard, the observed significant transitions were used to describe ethological patterns according to the different types of baits.

**Table 3:** Behaviours of baited attracted white sharks during ecotourism modified from Becerril-García et al.^22^ with its interpretation for management purposes in Guadalupe Island, Mexico.

Behaviour	Code	Description	Interpretation
Parading	PAR	The shark swims slowly around the bait. The distance between shark and bait usually ranges from 1 to 10 m	Curiosity display
Close Inspection	CLI	The shark swims close to the bait to a distance of < 1 m, without opening its mouth for consumption	Curiosity display
Horizontal strike	HS	The shark moves directly in the direction of the bait, with its mouth opening for capture at an angle < 45° in relation to the surface	Aggressive display
Vertical strike	VS	The shark moves from the deep, directly in the direction of the bait with its mouth open for capture, and at an angle of 46–90° in relation to the surface	Aggressive display
Bait caught	BAC	The shark closes its jaws with the bait inside	Stimuli reception
Feeding	FE	The shark swallows the bait after catching it	Stimuli reception
No feeding	NFE	The shark bites at the caught bait but releases it without consuming it	Stimuli reception
Buoy caught	BUO	The shark closes its jaws with the buoy inside its mouth	Stimuli reception

Additionally, the rate of bait capture and rate of consumption for each white shark category was determined through the following formulas:

$$BCR= \left[\frac{a}{\left(h+v\right)}\right]*100 \quad CoR= \left[\frac{b}{\left(h+v\right)}\right]*100$$

where *BCR* is the Bait Capture Rate; *CoR* is the Consumption Rate; *a* is the number of bait captures; *b* is the number of consumptions; *h* is the number of horizontal strikes; and *v* is the number of vertical strikes.

### Conditioning

The evaluation of the conditioning in the identified white sharks was carried out using a modified criteria generated from previous studies on shark behaviour^31–35,45^. For the purpose of this study, we define conditioning as associative learning obtained from a prolonged exposure to a stimulus associated with feeding behaviour. The number of visits per day, times of interaction and stimuli reception was registered for each photo identified white shark to determine the level of conditioning to the bait (Table 4). Stimulus reception was defined as the capture of the bait by a shark regardless its consumption. The criteria were intentionally modified to underestimate the learning capacity of the sharks by the assumption that white sharks have a similar or higher cognitive capacity than other studied elasmobranchs^1,31–33^. This decision was made due to the lack of studies regarding learning capacities of white sharks, differences between ecotourism effort in aggregation sites and its application for the monitoring of white sharks in the Guadalupe Island Biosphere Reserve^6,22^. In this way, the effect of baiting is described through a precautionary approach. These results were presented in histograms in order to obtain the proportions of identified white sharks and their respective degrees of conditioning.

**Table 4:** Criteria for the determination of conditioning degree of individual white sharks in Guadalupe Island during 2012–2014. Modified from Robbins^32^.

Conditioning degree	Description
No conditioning	Sharks visiting tourist boats less than three random days per season
I	Sharks visiting tourist boats more than three random days per season
II	Sharks visiting tourist boats between three to five random days per month
III	Sharks visiting the boats more than 5 days per month with the reception of at least one stimulus in such period
IV	Shark visiting the boats more than 5 days per month, with the reception of at least one stimulus, and interact more than 20 min per day

## Data Availability

The dataset regarding the monitoring of the evaluated white sharks is available from the corresponding author on request.
